# The Army, Hospital Governors and Resident Staffs

**Published:** 1915-05-08

**Authors:** 


### THE ARMY, HOSPITAL GOVERNORS AND RESIDENT STAFFS.

In the present national emergency every citizen no doubt has his own personal obligation to the cause of his country, and, in general, it is well for him to attend to this without looking too critically on the duty of his neighbour. In the Press we may claim some degree of exemption from this rule, or at least we may say that in our journalistic capacity our duty is to point out the opportunities for service which lie within the grasp of others. At the present moment very urgent demands are made on those responsible for the work of our voluntary hospitals, and in the consideration of these we wish to take our due share.

One of these demands reaches the hospitals indirectly, but it is none the less a very important and insistent request. We refer to the announcement made officially by the Director-General of the R.A.M.C. to the effect that an immediate supply of doctors ready to accompany the new Armies which are shortly to be sent to France is an urgent necessity. Unless the Medical Services are supplied the troops will not be able to take the field. No statement could be more authoritative or more unqualified. Now the hospitals in ordinary circumstances give resident appointments to the very type of man who, we are now told on the highest authority, is essential to the welfare of the soldiers and the safety of the nation. Give us, says the War Office, the younger men who can sustain the strain and hardships of an active campaign. The question is, What are the hospitals going to reply? It is not to the purpose to say that numbers of young doctors have already given up their appointments and have gone to the Front. The answer is that more are wanted. They are wanted in hundreds or even in larger figures. How in these circumstances can it be patriotic or even seemly to continue to attract the younger members of the profession to the service of the hospitals by the offer of increased salaries? Yet, if we may judge from advertisements in professional journals, this is exactly the policy which is being pursued. We suggest that such a proceeding needs justification, and we urge this consideration strongly on the attention of the governing body of every hospital.

It may be said that without a certain number of junior men as resident medical officers the work of the hospitals cannot be continued. We cannot accept this proposition. That the work must be continued is manifest, and we believe such a course is possible without detaining at home men who ought to be serving the country in the field. First, we would allow students in their fifth year to take resident appointments, and would count such service as part of their curriculum. In this way these men would be kept close to the original purpose of the fifth year—that is, they would be engaged in clinical work, and they would be the better fitting themselves to take service in the Army as soon as they have passed their final examinations. The leaders of the nation warn us to be prepared for a long struggle, and the need for Army doctors is not going to cease at the end of the present year. Then women residents should be encouraged so far as their numbers go, and a third source of supply may be found in men who are physically unfit for military duty. That these arrangements will be as efficient as those prevailing in normal times we do not say. But they may be fairly satisfactory, at least on one condition. This is that the visiting staffs sacrifice in some measure their personal comfort and interests in order that the work of the hospitals for the sick poor may not suffer at this time of national stress. Increased attendance and attention should supplement any possible deficiencies of the resident staff, and we believe this claim may be reasonably pressed in the public interest.

